# Research on the mechanism by which digital transformation peer effects influence innovation performance in emerging industries: A case study of China’s photovoltaic industry

**DOI:** 10.1371/journal.pone.0313615

**Published:** 2025-01-03

**Authors:** Aiqin Zhang, Xiaoqiang Guo, Wenjie Zhang, Zhangliang Liu

**Affiliations:** School of Economics and Management, North University of China, Taiyuan, China; Zhejiang University of Technology, CHINA

## Abstract

The exploration of digital transformation peer effects on the innovation performance of emerging industries is crucial for analyzing the underlying mechanisms of digital transformation, optimizing resource allocation among peer enterprises, and enhancing industrial competitiveness. This study empirically examines the influence of digital transformation peer effects on the innovation performance of the photovoltaic industry, using data from 150 photovoltaic companies listed in Shanghai and Shenzhen between 2011 and 2022. The study found that: (1) The digital transformation of the photovoltaic industry is influenced by regional and industry-specific peer effects. Regional peer effects in digital transformation have a positive impact on the innovation performance of the photovoltaic industry, while industry-specific peer effects exert a negative impact on innovation performance. Moreover, these effects exhibit dynamic persistence; (2) Further analysis of the transmission mechanism reveals that the digital transformation peer effect positively influences the innovation performance of the photovoltaic industry, primarily through the mediating role of enhanced absorptive capacity. Additionally, the level of marketization and executive tenure significantly moderate this relationship; (3) The study further investigates the photovoltaic industry within the context of subsidy policy implementation, firm types, and strategic pacing. The results indicate that the digital transformation peer effect on innovation performance is most pronounced for technology-intensive firms adopting an analytical strategy after the withdrawal of photovoltaic subsidies. For labor-intensive firms employing a defensive strategy, the peer effect is more significant before the withdrawal of subsidies. In contrast, the negative impact of industry-specific digital transformation peer effects on innovation performance is more evident in photovoltaic companies that pursue an offensive strategy; (4) The heterogeneity analysis reveals that the digital transformation peer effect on innovation performance is more significant for small-scale photovoltaic enterprises with state-owned property rights. In contrast, the peer effect negatively impacts innovation performance in large-scale photovoltaic enterprises. These findings provide theoretical insights and practical guidance for governments and enterprises in formulating digital transformation strategies for emerging industries.

## 1. Introduction

Amid the international shift toward reshaping the digital landscape, the digital transformation of China’s strategic emerging industries is critical to industrial upgrading and the country’s sustainable economic development. The 2023 report, *Overall Layout Plan for the Construction of Digital China*, issued by the Central Committee of the Communist Party of China and the State Council, identifies digitalization as a key engine for building a modern economic system. It emphasizes the need to further advance digital industrialization and industrial digitalization while promoting the deep integration of digital technologies with the real economy. Strategic emerging industries—such as next-generation information technology, new energy, and new materials—are essential for guiding the country’s future development. The deep integration of information technology with the real economy is expected to enhance the innovation and competitiveness of these industries, facilitating their advancement along the global value chain. Therefore, the digital transformation of emerging industries is expected to have a far-reaching impact on driving high-quality economic growth. As a leading sector in the development of strategic emerging industries, the photovoltaic (PV) industry spans the entire value chain, from upstream raw material production to midstream module manufacturing and system integration, and downstream construction and operation of PV power stations. The growth of this industry is pivotal to fostering and strengthening strategic emerging industries. With the global demand for renewable energy rising and China’s "dual carbon" goals (carbon peaking and carbon neutrality) in progress [1], the PV industry is experiencing unprecedented growth opportunities, yet it also faces challenges such as structural overcapacity, industrial homogeneity, and market competition. Digital transformation presents new opportunities for the PV industry, making it necessary not only to examine how digitalization supports its upgrading but also to explore the impact of digital transformation peer effects on innovation performance within the industry. This exploration is crucial for promoting the green and low-carbon development of the PV sector and optimizing the industrial value chain.

The peer effect of digital transformation refers to the process by which firms learn from, imitate, and influence one another during digital transformation. This effect arises not only from competitors within the same industry but also from partners, suppliers, and customers outside the sector [2]. Firstly, long-term stable development can be achieved by observing the successful practices of other firms within the same industry and learning from, optimizing, and adopting those practices [3]. Secondly, more mature technological standards and best practices are developed within the same industry, helping firms avoid duplication of effort, reduce innovation risks, and increase innovation efficiency [4]. Thirdly, the peer effect of digital transformation facilitates the sharing of resources, technologies, and market information among firms, enabling collaborative innovation and achieving mutually beneficial outcomes [5]. Finally, firms accelerate their innovation processes and enhance performance by imitating the innovative behaviors of peer firms. However, imitation itself does not constitute innovation; rather, it serves as a foundation from which firms can improve, differentiate, and develop original innovations [6]. Based on this, scholars widely acknowledge the facilitating role of the peer effect on firms’ innovative behavior. On the one hand, it is argued that external pressures from peers compel firms to engage in passive imitation, and these imitative pressures promote innovation through institutional isomorphism [7]. On the other hand, firms are believed to engage in active learning to reduce information asymmetry and gain a competitive advantage [8], thereby promoting innovative behavior.

However, existing studies have primarily focused on the impact of the digital transformation peer effect on the digital transformation of individual firms, without adequately exploring its mechanism on industrial innovation performance. Additionally, most research takes individual firms’ cognitive behavior as a mediating variable, yet fails to consider the management perspective of "cognition-comparison-action" in examining the peer effect. Therefore, this study uses China’s photovoltaic industry as a case to construct a theoretical model of "digital peer effect-learning ability-enterprise innovation performance." The aim is to explore the mechanism through which the digital transformation peer effect influences industrial innovation performance, providing a reference for emerging industries to leverage the peer effect in formulating strategic decisions and optimizing industrial policies.

## 2. Literature review

### 2.1. Digital transformation peer effect

Peer effects, initially studied in sociology and education, refer to the notion that an individual’s behavior is influenced not only by their own actions and decisions but also by those of their peers [9]. Related studies have defined cohort firms using various methods and confirmed the existence of peer effects. For example, some studies use industry classification as the criterion for constructing peer groups, verifying the presence of industry peer effects in areas such as share repurchase [10], corporate social responsibility [11], and investment behavior [12].Other scholars define peers based on geographic homogeneity to verify the existence of regional peer effects on firms’ innovation decisions [13], corporate social responsibility [14], and greenwashing behaviors [15]. Peers are also constructed using network-based criteria to confirm network peer effects in areas such as production networks [16], directors’ networks [17], and innovation input networks [18].

Research on the peer effect of enterprise digital transformation primarily focuses on its existence, mechanisms, influencing factors, and pathways. For instance, it is widely accepted that the cohort effect is a prevalent phenomenon in corporate behavior, and a digital cohort effect exists among listed companies in China [19]. A significant peer effect in enterprise digital transformation has been identified, with information transfer and dynamic competition contributing to its formation [20]. Additionally, the subjective motivation for imitation learning, along with factors such as network embeddedness, market competition, and environmental uncertainty, collectively enhance the peer effect of digital transformation [21].

### 2.2. The impact of peer effects on innovation performance

Mutual learning and imitation of R&D investment among peer firms significantly impact innovation output. At the micro level, the peer effect compensates for a firm’s lack of R&D experience; at the macro level, it functions similarly to innovation incentives, particularly for firms with limited R&D experience and those receiving government subsidies. Additionally, the peer effect varies across different market structures and positions, with a more pronounced effect on innovation performance in firms operating in competitive markets with lower market positions. For instance, research has shown that a learning orientation positively correlates with proactive innovation capabilities, and knowledge sharing among firms enhances these capabilities [22]. Furthermore, studies suggest that peer firms’ innovation influences the innovation behaviors of other firms through industrial cluster networks [23]. In recent years, the impact of digital transformation on corporate innovation performance has attracted widespread attention. As a critical external mechanism in digital transformation, peer effects play a pivotal role across various industries. Innovation-driven digital transformation not only alters companies’ production models and management practices but also enhances innovation performance through peer effects by facilitating information sharing and technology diffusion [24]. Existing studies indicate that digital transformation is influenced by both upstream and downstream enterprises within the supply chain [25], as well as by the digital interaction and collaboration among firms in the same region. Peer effects in digital transformation significantly improve innovation performance, particularly in technological innovation, product development, and production efficiency [26]. However, most current research focuses on individual firms or specific regions, lacking a systematic analysis of the overall industrial ecosystem and the interactive mechanisms between enterprises. Therefore, firms must account for various factors when developing innovation strategies to better leverage the peer effect in promoting innovation performance.

In summary, the existing literature offers extensive discussions on the existence and influencing factors of the peer effect in enterprise digital transformation. However, further investigation is required to understand the driving mechanism of peer effects on the innovation performance of emerging industries. Using the photovoltaic industry as an example, this paper analyzes the decision-making logic and action mechanisms of corporate core strategies in the digital era, considering industrial technology characteristics, economic attributes, policy dependence, and global competitiveness. The contributions of this paper are mainly reflected in: (1) verifying the existence of regional and industry peer effects in the digital transformation of the photovoltaic industry, and exploring the mechanisms through which these effects influence innovation performance; (2) addressing key strategic questions such as "what strategic pace to adopt" and "which direction technology- and labor-intensive firms should move toward" to drive rapid growth and performance improvement in the photovoltaic sector; and (3) in the context of the digital economy, investigating multiple pathways of influence and demonstrating the differential impacts of mediating and moderating variables, which further enriches research on the digital peer effects on innovation performance in emerging industries.

## 3. Theoretical analyses and research hypotheses

### 3.1. The existence of digital transformation peer effects

Social learning, competitive mimicry, and normative pressures among peer firms often lead focal firms to align their behaviors with those of their peers when making and implementing key decisions. The information and experiences shared within the peer network influence the behavior and decision-making processes of focal firms [27]. After observing significant capability gains and performance improvements resulting from digital transformation in peer firms within the same region, focal firms may pursue strategic transformation through active imitation and learning. Furthermore, when the digital transformation strategies of peer firms are positively evaluated by society, focal firms may adopt similar strategies to maintain or enhance organizational legitimacy.

Observing and learning from other firms within a cluster enhances the effectiveness of a firm’s behavior [28]. Management often references the practices of peer firms within the same industry when making significant decisions, and peer networks provide an efficient channel for learning and communication [29]. Peer firms in the cluster have improved resource utilization and collaborative processes through digital transformation, and this information is transmitted to the focal firm via the peer network. This transmission influences the focal firm’s perceptions and beliefs, prompting it to engage in active imitation, thereby improving its operations and decision-making processes.

Hypothesis 1: There are regional and industry peer effects in the digital transformation of the photovoltaic industry.

### 3.2. Mechanisms driving the digital transformation peer effects on the innovation performance of focal firms

For individual firms, the determination of the optimal course of action by photovoltaic companies is primarily based on both internal information and data regarding the actions of peer firms. When a firm’s executives fully represent shareholder interests and market information is complete [30], the influence of peer firms on decision-making is limited. In practice, however, photovoltaic firms often face the challenge of limited internal information, making external sources—such as peer firms, capital markets, local government data, and financial and legal intermediaries—critical references for strategic decisions [31]. The overlap in external information among firms within the same region, along with competition and confidentiality constraints, limits access to non-public information. This hinders firms’ ability to seize investment opportunities and mitigate market risks, thereby inhibiting their high-quality development.

The regional peer effect accelerates the diffusion and adoption of new technologies during digital transformation. First, when firms within a region adopt new technologies, other firms often follow the same path to maintain competitiveness, reducing the risks associated with independent exploration and enhancing innovation performance [32]. Second, knowledge sharing and technological exchange among firms are strengthened by the peer effect, promoting technological advancement both at the firm level and across the region’s industry [33]. The peer effect also increases competitive pressure among firms, driving improvements in the speed and capacity of innovation. Lastly, government support for digital transformation is amplified by the peer effect, providing stronger policy and resource backing for the innovative development of emerging industries [34].

Hypothesis 2: The peer effect of digital transformation in the same region of the photovoltaic industry positively affects the innovation performance of focal firms.

In clusters of firms, the theory of simulated competition suggests that firms maintain competitiveness through imitative learning [35]. Competitive interactions among firms influence investment decisions; for example, photovoltaic firms often mimic the investment timing and competitive strategies of industry peers to mitigate competitive risks or reduce pressures. Research indicates that the actions and decisions of firms regarding digital transformation interact as they face similar market environments and prospects. This explains why the digital transformation cohort effect can cause peer firms to collectively respond to policy opportunities, either displaying ’indifference’ by ignoring them or ’overreacting’ by flocking to them [36]. Additionally, when a photovoltaic firm engages in original innovation to gain a competitive edge, peer firms may quickly imitate and follow suit, hindering the innovator’s ability to improve innovation outputs, even with increased inputs. These digital peer effects within the same industry can diminish the motivation for original innovation, ultimately impeding high-quality development across the sector.

In the context of digital transformation within the same industry, the peer effect can have multiple impacts on the innovation of focal firms. First, the peer effect can easily lead to technological path dependency [37]. When many firms adopt similar digital technologies or platforms, focal firms tend to choose widely used technological paths, which may limit their innovation space, increase adjustment costs, and weaken their capacity for independent innovation. Second, the peer effect may induce innovation inertia. After peer firms achieve success through digital transformation, focal firms often resort to imitation rather than pursuing original innovation, lacking the motivation for differentiated innovation [38]. Third, the collective transformation of peer firms can dilute the innovation resources of focal firms [39]. To remain competitive, focal firms must allocate resources across multiple areas, reducing their focus on specific innovation directions and thereby diminishing innovation performance. Finally, the peer effect may exacerbate the innovation risks faced by focal firms [40]. Uncertain technological and market environments increase innovation pressures; while imitating the successful paths of peers may help reduce short-term risks, it could ultimately result in lost opportunities for independent innovation and long-term competitive advantage.

Hypothesis 3: The peer effect of digital transformation in the same photovoltaic industry sector negatively affects focal firms’ innovation performance.

**Fig 1:**
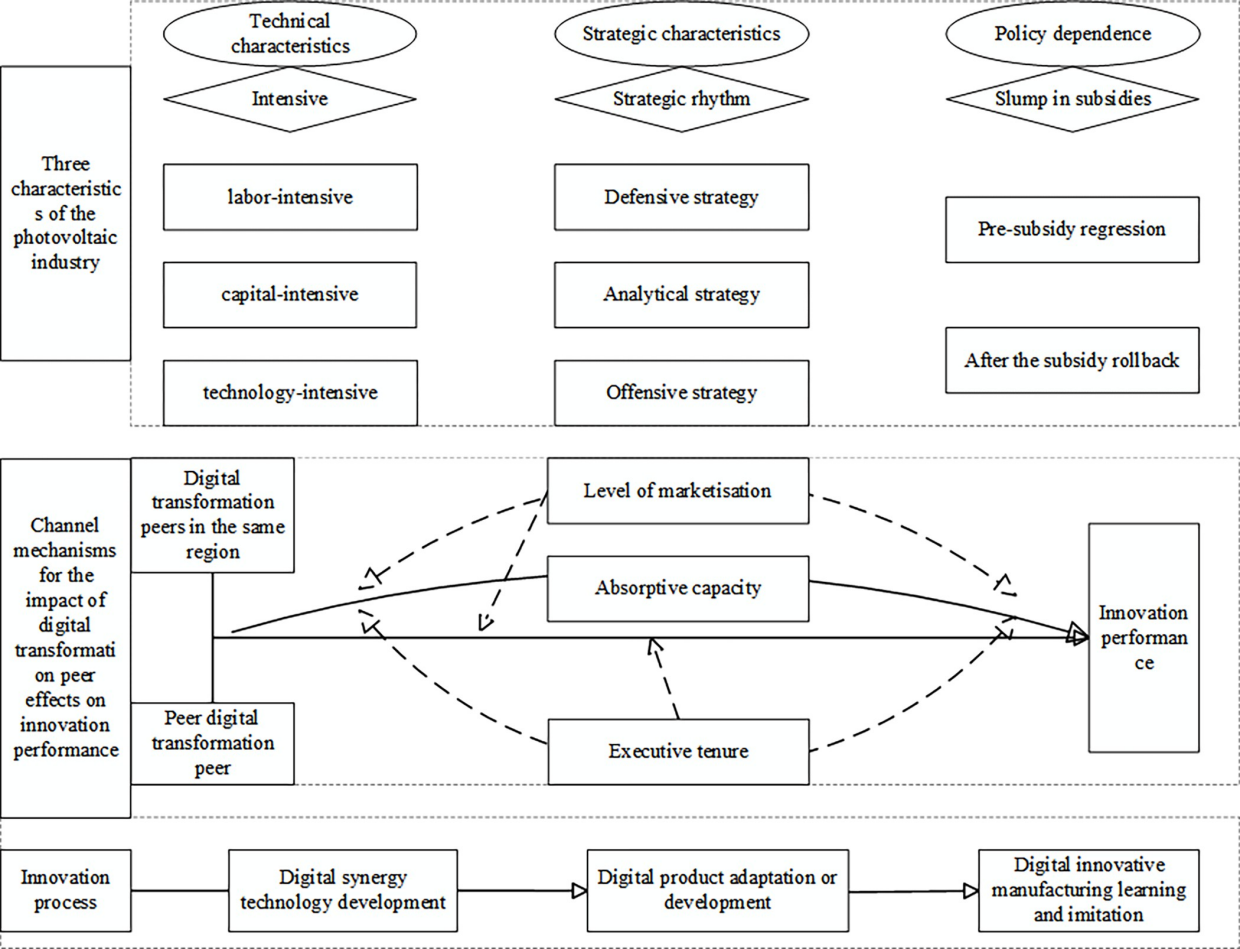
Main logical framework.

Fig 1 presents the analytical framework used in this study.

## 4. Research design

### 4.1. Sample selection and data sources

This study uses data from 150 photovoltaic (PV) companies listed on China’s Shanghai and Shenzhen A-share markets from 2011 to 2022 as the initial research sample. The following steps were taken: (1) financial companies were excluded; (2) firms with ST and *ST status were excluded; (3) firms with IPOs during the study period were excluded; (4) only samples with at least five consecutive years of complete data were retained; (5) to reduce the impact of outliers, all micro-level continuous variables were winsorized at the 1% and 99% levels. Data on enterprise digital transformation cohorts were primarily obtained through text analysis of annual reports from listed companies, while the remaining data were sourced from the Cathay Pacific Database (CSMAR), Wande Database (WIND), and the China Research Data Service Platform (CNRDS).

### 4.2. Variable definition and measurement

1. Photovoltaic industry innovation performance (ip) is primarily measured using the number of patents granted and patent applications, as established in existing studies [41]. Therefore, this paper assesses the innovation performance of photovoltaic companies based on the number of patent applications. These patent applications include invention patents, utility models, and design patents.
2. Peer effects of digital transformation (peer_a; peer_i) are often measured using technologies such as artificial intelligence, blockchain, cloud computing, big data, and digital business models as the foundational lexicon for defining digital transformation [42,43]. Since the peer effect of digital transformation combines the concepts of digital transformation and peer effect [44], this paper follows the approach of defining peer firms as those registered in the same province or operating in the same industry as the focal firm [45]. The degree of digital transformation among same-region and same-industry peers, excluding the focal firm, is used as a measure of peer digital transformation. The steps are as follows: First, a text search, matching, and word frequency count of keywords related to "digital transformation" is conducted to construct an enterprise digital transformation index. Python software is then used to calculate the word frequency of digital transformation terms for each company, with the values logarithmized. Second, the average digital transformation level of peer firms in the same region or industry, excluding the focal firm, is calculated.
3. Absorptive capacity (ac) is characterized in this paper by R&D intensity. To account for differences in firm size, the ratio of R&D investment to operating revenue is used as the measure of absorptive capacity [46,47].
4. Level of marketization (market) and executive tenure (ceo) are key variables in this study. The level of marketization encompasses factors such as the relationship between government and market forces, the development of the non-state economy, the advancement of product and factor markets, the growth of market intermediary organizations, and the legal and institutional environment. The marketization indicator is derived by applying the calculation methods used in previous studies [48]. For executive tenure, executives are classified into roles such as chairman (vice), general manager (vice), president (vice), secretary to the chairman, assistant to the general manager, and key directors, based on their functional roles and the nature of the firm’s strategic behaviors. The average years of service of the executive team is used as the measure of executive tenure.
5. Control variables. Following the approach of existing literature [49,50], this paper includes the gearing ratio (lev), cash flow ratio (cashflow), growth rate of operating income (growth), number of directors (board), proportion of independent directors (indep), and the proportion of shares held by the top five shareholders (top5) as control variables. The definitions and symbols of these variables are presented in Table 1.

**Table 1:** Definition of variables.

Variable type	Variable name	Variable symbols	Description of variables
Explanatory variables	Digital transformation	dt	The total frequency of words related to digital transformation in corporate annual reports
Core explanatory variables	Photovoltaic industry innovation performance	ip	Number of patent applications divided by 1000
	Peer effect of digital transformation in the same region	peer_a	Mean value of digital transformation of other companies in the same province
	Peer effect of digital transformation in the same industry	peer_i	Average value of digital transformation of other companies in the same industry
Intermediary variable	Absorptive capacity	ac	R&D expenditure to operating income
Moderator variables	Level of marketisation	market	Marketisation index
	Executive tenure	ceo	Mean value of executive tenure
Control variables	Gearing ratio	lev	Total liabilities at year-end divided by total assets at year-end
	Cashflow ratio	cashflow	Net cash flows from operating activities divided by total assets
	Revenue growth rate	growth	Operating income for the year divided by operating income for the previous year minus 1
	Number of directors	board	The number of board members is taken as a natural logarithm
	Proportion of independent directors	indep	Independent directors divided by number of directors
	Shareholding ratio of top five shareholders	top5	Number of shares held by top five shareholders divided by total number of shares

### 4.3. Model setting

1. The following model examines the direct impact of digital transformation peer effects on the innovation performance of photovoltaic firms.
$$ip_{it}=\alpha_{0}+\alpha_{1}peer\_a_{it}+\alpha_{2}controls_{it}+\alpha_{3}year_{t}+\alpha_{4}id_{i}+\varepsilon$$ (1)
$$ip_{it}=\beta_{0}+\beta_{1}peer\_i_{it}+\beta_{2}controls_{it}+\beta_{3}year_{t}+\beta_{4}id_{i}+\varepsilon$$ (2)
  In this model, ip_it_ represents the innovation performance of firm i in year t, while peer_a_it_ and peer_i_it_ represent digital transformation in the same industry and the same region, respectively. controls_it_ denotes the corresponding control variables, with individual and time-fixed effects included in the model. If the coefficients α_1_ and β_1_ are significant, it indicates that the digital transformation peer effect enhances innovation performance. ε represents the random error term.
2. The following model investigates the mechanism through which digital transformation peer effects influence the innovation performance of photovoltaic firms.
$$M_{it}=\gamma_{0}+\gamma_{1}peer\_a_{it}+\gamma_{2}controls_{it}+\gamma_{3}year_{t}+\gamma_{4}id_{i}+\varepsilon$$ (3)
$$M_{it}=\phi_{0}+\phi_{1}peer\_i_{it}+\phi_{2}controls_{it}+\phi_{3}year_{t}+\phi_{4}id_{i}+\varepsilon$$ (4)
$$ip_{it}=\lambda_{0}+\lambda_{1}peer\_a_{it}+\lambda_{2}M_{it}+\lambda_{3}controls_{it}+\lambda_{4}year_{t}+\lambda_{5}id_{i}+\varepsilon$$ (5)
$$ip_{it}=\mu_{0}+\mu_{1}peer\_i_{it}+\mu_{2}M_{it}+\mu_{3}controls_{it}+\mu_{4}year_{t}+\mu_{5}id_{i}+\varepsilon$$ (6)
  Where M_it_ represents the mediating variable, absorptive capacity.
3. The following model investigates the moderating effects of marketization level and executive tenure.
$$\begin{array}{l} ip_{it}=\eta_{0}+\eta_{1}peer\_a_{it}+\eta_{2}X_{it}+\eta_{3}peer\_a_{it}\times X_{it}+\eta_{4}controls_{it}\\ +\eta_{5}year_{t}+\eta_{6}id_{i}+\varepsilon \end{array}$$ (7)
$$\begin{array}{l} ip_{it}=\sigma_{0}+\sigma_{1}peer\_i_{it}+\sigma_{2}X_{it}+\sigma_{3}peer\_i_{it}\times X_{it}+\sigma_{4}controls_{it}\\ +\sigma_{5}year_{t}+\sigma_{6}id_{i}+\varepsilon \end{array}$$ (8)
$$\begin{array}{l} M_{it}=\phi_{0}+\phi_{1}peer\_a_{it}+\phi_{2}X_{it}+\phi_{3}peer\_a_{it}\times X_{it}+\phi_{4}controls_{it}\\ +\phi_{5}year_{t}+\phi_{6}id_{i}+\varepsilon \end{array}$$ (9)
$$\begin{array}{l} M_{it}=\omega_{0}+\omega_{1}peer\_i_{it}+\omega_{2}X_{it}+\omega_{3}peer\_i_{it}\times X_{it}+\omega_{4}controls_{it}\\ +\omega_{5}year_{t}+\omega_{6}id_{i}+\varepsilon \end{array}$$ (10)
$$\begin{array}{l} ip_{it}=\theta_{0}+\theta_{1}peer\_a_{it}+\theta_{2}X_{it}+\theta_{3}peer\_a_{it}\times X_{it}+\theta_{4}M_{it}\\ +\theta_{5}M_{it}\times X_{it}+\theta_{6}controls_{it}+\theta_{7}year_{t}+\theta_{8}id_{i}+\varepsilon \end{array}$$ (11)
$$\begin{array}{l} ip_{it}=\zeta_{0}+\zeta_{1}peer\_i_{it}+\zeta_{2}X_{it}+\zeta_{3}peer\_i_{it}\times X_{it}+\zeta_{4}M_{it}\\ +\zeta_{5}M_{it}\times X_{it}+\zeta_{6}controls_{it}+\zeta_{7}year_{t}+\zeta_{8}id_{i}+\varepsilon \end{array}$$ (12)
  Where X_it_ represents the moderating variable, either marketization level or executive tenure.

### 4.4. Descriptive statistics

Table 2 reports the innovation performance of photovoltaic firms, with a maximum value of 4.463, a minimum value of 0, a mean of 0.101, and a standard deviation of 0.304, measured in thousands. This indicates that the overall level of innovation performance among China’s photovoltaic enterprises is relatively low, with significant fluctuations across firms. The mean value of the peer effect of digital transformation within the same region is 1.377, with a maximum of 4.457 and a minimum of -0.294, showing a greater dispersion in regional peer effects compared to firm-level performance. The industry peer effect of digital transformation has a mean value of 1.191, with a maximum of 4.055 and a minimum of -3.858, exhibiting even greater dispersion than the regional peer effect. The mean value of firms’ absorptive capacity is 0.0400, with a maximum of 0.485 and a minimum of -0.300, indicating more concentrated and stable data. This descriptive statistical information highlights the significant variability in the key variables.

**Table 2:** Descriptive statistics of main variables.

Variables	Sample size	Mean value	Standard deviation	Minimum value	Medium value	Maximum
dt	1800	4.857	15.35	-105	1	128
ip	1800	0.101	0.304	0	0.0100	4.463
peer_a	1800	1.377	0.558	-0.294	1.434	4.457
Peer_i	1800	1.191	0.810	-3.858	1.073	4.055
ac	1800	0.0400	0.0530	-0.300	0.0350	0.485
lev	1800	0.416	0.342	-2.076	0.471	1.652
cashflow	1800	0.0250	0.215	-2.186	0.0430	1.001
growth	1800	0.106	1.286	-10.49	0.108	10.89
board	1800	2.151	0.249	0.611	2.197	4.204
indep	1800	0.361	0.0850	-0.607	0.333	0.636
top5	1800	0.600	0.196	0.0850	0.592	1.866

## 5. Rusults and discussion

### 5.1. Existence test for the peer effect of digital transformation in the photovoltaic industry

#### 5.1.1. Benchmark regression

Firstly, the peer effect of digital transformation in the photovoltaic industry is examined, with the results presented in Table 3. Columns (1) and (3) show the regression results of the impact of peer firms’ digital transformation on the focal firms without control variables, while columns (2) and (4) include control variables. The results in column (2) indicate that the peer effect of digital transformation within the same region has a coefficient of 2.340 and is significant at the 1% level. Similarly, the results in column (4) reveal that the peer effect of digital transformation within the photovoltaic industry has a coefficient of 6.560 and is also significant at the 1% level. These findings demonstrate a significant peer effect in the digital transformation of the photovoltaic industry, where the digital transformation of focal firms increases as the digital transformation of peer firms intensifies. Thus, research hypothesis 1 is supported. Existing literature primarily focuses on the impact of digital transformation peer effects on corporate high-quality development, innovation investment, and carbon emissions. This study, however, concentrates on the influence of digital transformation peer effects on innovation performance in emerging industries, thereby broadening the research perspective. Unlike prior research that focuses on the innovation outcomes of individual firms’ digital transformation activities, this study integrates the externalities and peer effects of digital transformation into the analytical framework, revealing the internal mechanisms through which firms enhance innovation performance via peer interactions in the context of digital transformation. While existing studies mainly focus on the peer effects within industries such as manufacturing, services, and distribution, the unique contribution of this research lies in its first systematic analysis of peer effects in promoting innovation in the emerging photovoltaic industry in China. This work expands the breadth and depth of digital transformation research.

**Table 3:** Results of the digital transformation peer effect test.

	(1)	(2)	(3)	(4)
	dt	dt	dt	dt
peer_a	2.254***	2.340***		
	(2.66)	(2.74)		
peer_i			6.841***	6.560***
			(8.05)	(7.85)
growth		1.699***		1.520**
		(2.85)		(2.57)
board		4.499***		2.543**
		(3.42)		(2.05)
indep		-1.610		-2.079
		(-0.35)		(-0.48)
lev		-0.369		-0.422
		(-0.31)		(-0.40)
top5		-5.841***		-2.766
		(-3.07)		(-1.56)
cashflow		7.388***		8.553***
		(4.05)		(5.25)
_cons	-1.471	-6.408*	-3.774***	-6.051
	(-1.11)	(-1.76)	(-2.76)	(-1.63)
N	1800	1800	1800	1800
R2	0.042	0.091	0.144	0.181
adj. R2	0.036	0.081	0.138	0.173
F	8.931	8.817	14.123	11.714

Note:

***, **, and * indicate significance at the 1%, 5%, and 10% levels, respectively. T-values are shown in parentheses, and all regression coefficients’ standard errors are cluster-processed at the firm level. The following tables follow the same format.

The regression results of the peer effects of the two digital transformations on the innovation performance of the photovoltaic industry are shown in Table 4. Columns (1) and (3) present the baseline regression results without control variables, while columns (2) and (4) include control variables. According to the estimation results in column (1), the regression coefficient of the digital peer effect on innovation performance within the same region is 0.045 and significantly positive at the 1% level. This indicates that the peer effect of digital transformation enhances innovation performance in the photovoltaic industry, supporting Hypothesis 2. In column (3), the regression coefficient of the peer effect of digital transformation within the same industry on innovation performance is -0.022 and is significantly negative at the 1% level. This suggests that the peer effect of digital transformation within the photovoltaic industry sector inhibits innovation performance, confirming Hypothesis 3.

**Table 4:** Digital transformation peer effects and innovation performance.

	(1)	(2)	(3)	(4)
	ip	ip	ip	ip
peer_a	0.045***	0.086***		
	(2.72)	(3.66)		
peer_i			-0.022***	-0.019***
			(-3.97)	(-3.88)
lev		0.139***		0.095***
		(4.97)		(5.00)
cashflow		0.075***		0.070***
		(3.41)		(3.01)
growth		-0.006		-0.002
		(-1.42)		(-0.44)
board		-0.015		0.001
		(-0.50)		(0.02)
indep		0.261***		0.214**
		(2.65)		(2.29)
top5		0.021		0.025
		(0.59)		(0.67)
_cons	-0.003	-0.148**	0.043***	-0.079
	(-0.21)	(-2.20)	(4.52)	(-1.19)
N	1800	1800	1800	1800
R2	0.040	0.065	0.039	0.054
adj. R2	0.034	0.055	0.033	0.045
F	5.279	5.494	5.376	6.301

#### 5.1.2. Robustness test

1. Explanatory variables are lagged by one period. The benchmark regression in this paper focuses on the impact of digital transformation peer effects on the innovation performance of focal firms. Generally, group behaviors and decisions exert a more significant influence on individuals, whereas individual actions rarely impact the group. However, when the focal firm is a leading entity within the peer group, the progressive demonstration effect of its digital transformation is more likely to inspire emulation by other firms, thereby strengthening the mechanism through which digital transformation drives innovation. To address potential endogeneity caused by mutual causation, the explanatory variables are lagged by one period. In Table 5, the results indicate that the digital transformation peer effect with a one-period lag continues to have a significant impact on current innovation performance. This suggests that the peer effect of digital transformation within the same region and industry persists for at least one year, confirming the robustness of the previous regression results.
2. Robustness tests were conducted using Tobit regression. Tobit regression was applied due to the large number of zero values for the explanatory variable, innovation performance, which is characterized by left truncation. The results in column (1) of Table 6 show that the peer effect of digital transformation within the same region remains significantly positively correlated with innovation performance. Similarly, the results in column (2) indicate that the peer effect of digital transformation within the same industry remains significantly negatively correlated with innovation performance, confirming the robustness of the benchmark regression results.
3. Instrumental variables approach. The mean values of digital transformation for other enterprises in the same province as the focal enterprise were selected, and peer indicators of digital transformation within the same region, measured by the frequency of digital transformation terms in Management Discussion and Analysis (MD&A) sections, were used as instrumental variables. Additionally, digital transformation trends within the same region and industry were analyzed by selecting the mean values of digital transformation for other enterprises in the same industry as the focal enterprise and using peer indicators of digital transformation in the same sector, also measured by the frequency of digital transformation terms in management discussions, as instrumental variables.

**Table 5:** Explanatory variables lagged one period test results.

	(1)	(2)	(3)	(4)
	ip	ip	ip	ip
lev	0.139***	0.162***	0.095***	0.116***
	(4.97)	(4.79)	(5.00)	(4.92)
cashflow	0.075***	0.096***	0.070***	0.087***
	(3.41)	(3.26)	(3.01)	(2.84)
growth	-0.006	-0.006	-0.002	-0.002
	(-1.42)	(-1.16)	(-0.44)	(-0.41)
board	-0.015	-0.013	0.001	0.001
	(-0.50)	(-0.37)	(0.02)	(0.04)
indep	0.261***	0.310**	0.214**	0.260**
	(2.65)	(2.55)	(2.29)	(2.25)
top5	0.021	0.028	0.025	0.029
	(0.59)	(0.69)	(0.67)	(0.69)
peer_a	0.086***			
	(3.66)			
L.peer_a		0.081***		
		(3.25)		
peer_i			-0.019***	
			(-3.88)	
L.peer_i				-0.023***
				(-4.34)
_cons	-0.148**	-0.169**	-0.079	-0.095
	(-2.20)	(-2.05)	(-1.19)	(-1.18)
N	1800	1650	1800	1650
R2	0.065	0.061	0.054	0.054
adj. R2	0.055	0.051	0.045	0.044
F	5.494	5.095	6.301	5.995

**Table 6:** Tobit test results.

	(1)	(2)
	ip	ip
peer_a	0.185***	
	(0.013)	
peer_i		-0.120***
		(0.011)
lev	0.001	-0.024
	(0.031)	(0.032)
cashflow	0.083*	0.131**
	(0.050)	(0.052)
growth	0.029***	0.027***
	(0.007)	(0.007)
board	0.061	0.031
	(0.044)	(0.047)
indep	0.432***	0.511***
	(0.126)	(0.133)
top5	0.054	-0.004
	(0.062)	(0.064)
_cons	-0.588***	-0.399***
	(0.133)	(0.138)
sigma_u	0.342***	0.350***
	(0.023)	(0.023)
sigma_e	0.191***	0.197***
	(0.004)	(0.004)
N	1800.000	1800.000

Theoretically, the level of digital development in the region where the company operates affects the firm’s own degree of digitization, meeting the correlation condition. The digital development level of firms in the same industry within the region does not directly affect firms’ innovation performance, satisfying the exogeneity condition. Based on existing research literature [51], the mean value of the digitization level of firms in the same industry and year, within the same province, excluding the focal firm, was used as the instrumental variable. The 2SLS method was then employed for re-estimation. Columns (1) and (2) in Table 7 present the results of the corresponding tests, with first-stage regression coefficients significantly positive at the 1% level, passing tests for under-identification and weak instruments, thus supporting the validity of the selected instrumental variables. The second-stage regression coefficients also remain significantly positive at the 1% level, confirming the robustness and reliability of the conclusions after the selection of instrumental variables.

**Table 7:** Instrumental variable method test results.

	2sls	
	ip	ip
GJ1	0.107***	
	(0.020)	
GJ2		-0.025***
		(0.007)
lev	0.152***	0.154***
	(0.025)	(0.023)
cashflow	0.073***	0.072***
	(0.021)	(0.022)
growth	-0.005	-0.005
	(0.004)	(0.004)
board	-0.012	-0.011
	(0.026)	(0.028)
indep	0.282***	0.284***
	(0.095)	(0.094)
top5	-0.001	-0.004
	(0.034)	(0.034)
_cons	-0.163**	-0.170**
	(0.070)	(0.078)
N	1800.000	1800.000
r2	0.061	0.061
r2_a	0.057	0.057

### 5.2. the driving mechanism of the digital transformation cohort effect of the photovoltaic industry test

#### 5.2.1. The mediating role of absorptive capacity

This section further investigates the underlying mechanisms of the main effect, focusing on two key questions to identify potential channels of influence. First, does the peer effect of digital transformation within the same region enhance the innovation performance of the photovoltaic industry through absorptive capacity? Second, does the peer effect of digital transformation within the same industry mitigate its negative impact on the innovation performance of the photovoltaic industry through absorptive capacity? Additionally, this paper explores how the peer effect of digital transformation influences the efficiency of corporate innovation inputs and outputs, ultimately promoting the high-quality development of enterprises.

Table 8 presents the empirical results of Models 1 through 3, which show stepwise regression results of the mediating effect of absorptive capacity. Model 1 reflects the impact of the digital transformation peer effect on the innovation performance of focal firms within the same region and industry. Model 2 examines the effect of the peer effect on the mediating variable, absorptive capacity. Model 3 builds upon Model 1 by including absorptive capacity as a mediating variable. The regression results in the first column confirm that the peer effect of digital transformation within the same region is positively associated with innovation performance, with an estimated coefficient of 0.094. Similarly, the results show that the peer effect of digital transformation within the same industry is significantly negatively associated with innovation performance, with an estimated coefficient of -0.027. The second column demonstrates a significant positive relationship between the peer effect of digital transformation within the same region and absorptive capacity, with estimated coefficients of 0.007 and 0.014. This suggests that the impact of the same-region digital transformation peer effect on innovation performance is further strengthened by the mediating role of absorptive capacity. In contrast, the negative impact of the same-industry peer effect on innovation performance is reduced, but remains negative.

**Table 8:** Results of the mediation effect test.

	Model 1	Model 2	Model 3
	ip	ac	ip
peer_a	0.094***	0.007*	0.091***
	(3.83)	(1.79)	(3.79)
peer_i	-0.027***	0.014***	-0.030***
	(-4.67)	(3.42)	(-4.52)
ac			0.442***
			(4.31)
lev	0.139***	-0.017***	0.153***
	(4.98)	(-3.79)	(5.36)
cashflow	0.070***	0.015*	0.076***
	(3.22)	(1.86)	(3.61)
growth	-0.005	-0.003	-0.008***
	(-1.22)	(-1.46)	(-3.01)
board	-0.006	-0.007	-0.005
	(-0.22)	(-1.53)	(-0.19)
indep	0.262***	0.011	0.261***
	(2.65)	(0.96)	(2.63)
top5	0.009	-0.038***	0.029
	(0.26)	(-4.44)	(0.80)
_cons	-0.147**	0.062***	-0.176**
	(-2.22)	(4.33)	(-2.51)
N	1800	1800	1800
R2	0.069	0.089	0.072
adj. R2	0.059	0.079	0.062
F	5.736	6.950	5.402

#### 5.2.2. The moderating role of the level of marketisation and executive tenure

The moderating effects of marketization level and executive tenure are tested as shown in Table 9. Models 1 through 6 present the empirical results of Eqs (7) through (12). The results for market uncertainty are reflected in Models 1, 3, and 5. Model 1 adds the interaction term between marketization level and absorptive capacity based on Model 3 in Table 7. Model 3 includes the interaction between marketization level and the peer effect of digital transformation within the same region, based on Model 2 in Table 7. Model 5 introduces the interaction between marketization level and the peer effect of digital transformation within the same industry, also based on Model 2 in Table 7. The results for executive tenure are shown in Models 2, 4, and 6. Model 2 adds the interaction between executive tenure and absorptive capacity based on Model 3 in Table 7. Model 4 includes the interaction between executive tenure and the peer effect of digital transformation within the same region, based on Model 2 in Table 7. Finally, Model 6 introduces the interaction between executive tenure and the peer effect of digital transformation within the same industry, also based on Model 2 in Table 7.

**Table 9:** Test results for marketization level and executive tenure regulation.

	Model 1	Model 2	Model 3	Model 4	Model 5	Model 6
	ip	ip	ac	ac	ac	ac
peer_a	0.098***	0.109***	-0.008	0.016***		
	(3.85)	(4.06)	(-1.11)	(3.44)		
peer_i	-0.030***	-0.032***			-0.014*	0.018***
	(-4.40)	(-4.27)			(-1.78)	(3.86)
ac*market	0.045***					
	(3.94)					
ac*ceo		0.049***				
		(3.42)				
peer_a*market			0.002***			
			(3.60)			
peer_a*ceo				-0.000		
				(-0.26)		
peer_i*market					0.003***	
					(4.31)	
peer_i*ceo						0.000***
						(5.00)
lev	0.171***	0.195***	-0.023***	-0.027***	-0.024***	-0.030***
	(5.33)	(5.25)	(-4.33)	(-4.52)	(-5.39)	(-5.09)
cashflow	0.085***	0.191***	0.013*	0.010	0.013	0.008
	(3.76)	(4.21)	(1.67)	(0.80)	(1.59)	(0.60)
growth	-0.009***	-0.014***	-0.002	-0.000	-0.002	-0.001
	(-3.12)	(-3.49)	(-0.98)	(-0.20)	(-0.95)	(-0.30)
board	-0.009	0.060*	0.001	0.010	-0.000	0.005
	(-0.29)	(1.84)	(0.24)	(1.04)	(-0.05)	(0.61)
indep	0.251**	0.709***	0.009	0.022	0.005	0.021
	(2.45)	(3.24)	(0.83)	(0.76)	(0.41)	(0.77)
top5	0.021	0.010	-0.042***	-0.062***	-0.030***	-0.043***
	(0.57)	(0.23)	(-4.74)	(-5.67)	(-3.74)	(-4.27)
_cons	-0.170**	-0.484***	0.058***	0.045*	0.053***	0.046*
	(-2.29)	(-3.67)	(3.88)	(1.69)	(3.60)	(1.78)
N	1800	1800	1800	1800	1800	1800
R2	0.072	0.087	0.064	0.068	0.111	0.123
adj. R2	0.061	0.074	0.053	0.056	0.101	0.111
F	5.093	4.930	8.226	6.134	6.023	6.098

The results from Models 1 and 2 show that the estimated coefficients of the interaction terms between the mediating variable absorptive capacity and the moderating variables marketization level and executive tenure are positive and significant, indicating that a higher level of marketization or longer executive tenure enhances the positive impact of absorptive capacity on innovation performance in the photovoltaic industry. The results from Models 3 and 4 demonstrate that the interaction term between the peer effect of digital transformation within the same region and marketization level is positive and significant at the 1% level, while the interaction term between the peer effect of digital transformation within the same region and executive tenure is not significant. The results from Models 5 and 6 indicate that the interaction terms between the peer effect of digital transformation within the same industry and both marketization level and executive tenure are positive and significant at the 1% level.

These findings suggest that as marketization levels increase or executive tenure lengthens, the positive effect of digital transformation peer effects—both within the same region and industry—on innovation performance becomes stronger. However, the moderating effect of executive tenure on the relationship between the peer effect of digital transformation within the same region and absorptive capacity is not significant.

#### 5.2.3. Impact of different intensive type choices on innovation performance

To better guide the photovoltaic industry in positioning itself for development, this paper introduces firm-intensity types to analyze the cohort effect within the industry and determine which type of intensity is more conducive to enhancing innovation performance. Firm-intensity types refer to a company’s high reliance on a specific production factor in its processes and can be categorized into three types: labor-intensive, capital-intensive, and technology-intensive. Each type has distinct characteristics and strengths, leading to different developmental responses.

Table 10 presents the results of tests on different firm-intensity types within the photovoltaic sector. It was found that the peer effect of digital transformation within the same region had the most significant positive impact on innovation performance for labor-intensive and technology-intensive firms. In contrast, the impact for capital-intensive firms was not substantial. However, the peer effect of digital transformation within the same industry had a significant negative impact on innovation performance for technology-intensive firms. These findings suggest that the direction of digital transformation should be tailored to the firm’s factor dependence. Labor- and technology-intensive firms within the same region benefit more from the peer effect of digital transformation, while the significant negative peer effect experienced by technology-intensive firms within the same industry requires attention, so that appropriate measures can be taken to mitigate the issue.

**Table 10:** Test results of different photovoltaic firms’ intensive type selection.

	labour-intensive		capital-intensive		technology-intensive	
	ip	ip	ip	ip	ip	ip
peer_a	0.144***		0.014		0.145***	
	(3.56)		(1.12)		(2.98)	
peer_i		-0.032		-0.050		-0.063***
		(-1.62)		(-1.07)		(-4.91)
lev	0.210***	0.160***	0.052***	0.065***	0.258***	0.192***
	(3.82)	(3.41)	(3.16)	(3.59)	(3.81)	(4.07)
cashflow	0.067	0.025	0.016	0.022	0.240***	0.264***
	(0.83)	(0.32)	(0.29)	(0.38)	(4.48)	(4.46)
growth	0.005	0.018**	0.004	0.003	-0.032***	-0.018***
	(0.59)	(2.53)	(0.48)	(0.39)	(-4.56)	(-2.76)
board	0.006	0.013	0.019	0.006	0.080**	0.185***
	(0.10)	(0.20)	(0.48)	(0.14)	(2.03)	(4.58)
indep	0.019	0.068	-0.022	-0.009	0.829***	0.775***
	(0.09)	(0.30)	(-0.34)	(-0.15)	(3.41)	(3.24)
top5	0.147	0.224**	-0.025	-0.027	0.007	0.014
	(1.49)	(1.99)	(-0.69)	(-0.76)	(0.12)	(0.23)
_cons	-0.290	-0.262	0.004	-0.002	-0.606***	-0.630***
	(-1.64)	(-1.37)	(0.04)	(-0.02)	(-3.32)	(-3.68)
N	395	395	488	488	917	917
R2	0.163	0.127	0.072	0.074	0.082	0.078
adj. R2	0.119	0.081	0.036	0.038	0.064	0.059
F	2.032	2.185	2.814	2.909	3.387	4.206

#### 5.2.4. Impact of different strategic tempo choices on innovation performance

With the rapid development of emerging industries, corporate innovation and change are accelerating. Companies must continuously adapt to market demands by adjusting their development strategies and business models. The flexibility and adaptability of strategic pacing are crucial in this process [52]. Strategic pacing reflects a company’s aggressiveness in strategic planning and development, encompassing various approaches such as passive-conservative, proactive, and risk-taking. This paper introduces the concept of strategic rhythms to explore peer effect decision-making, classifying corporate strategic rhythms into three categories: defensive, analytical, and offensive.

Table 11 presents the results of the tests for different strategic pacing choices. The study shows that when firms adopt analytical strategies, the positive impact of the digital transformation peer effect on innovation performance within the same region is most significant at the 1% level, while the negative impact of the same-industry peer effect on innovation performance in emerging industries is also most significant at the 1% level. For firms adopting defensive strategies, the positive impact of the regional peer effect on innovation performance is significant at the 10% level, with no substantial impact observed within the same industry. Conversely, when firms adopt offensive strategies, the negative impact of the regional peer effect on innovation performance is significant at the 10% level, and the negative impact of the same-industry peer effect is similarly substantial at the 10% level.

**Table 11:** Test results for different strategic pacing options.

	defensive strategy		analytical strategy		offensive strategy	
	ip	ip	ip	ip	ip	ip
peer_a	0.026*		0.096***		0.070	
	(0.60)		(2.82)		(1.19)	
peer_i		-0.002		-0.029***		-0.028*
		(-0.14)		(-3.06)		(-1.30)
lev	0.005	-0.004	0.294***	0.270***	0.122**	0.116*
	(0.13)	(-0.13)	(3.92)	(3.90)	(2.05)	(1.96)
cashflow	0.011	0.017	0.658**	0.638**	0.355	0.317
	(0.40)	(0.63)	(2.55)	(2.46)	(1.51)	(1.30)
growth	0.018	0.021	-0.012**	-0.011*	0.053*	0.054*
	(0.78)	(0.85)	(-1.97)	(-1.72)	(1.67)	(1.76)
board	0.151**	0.156*	-0.144**	-0.166**	0.461**	0.486**
	(2.00)	(1.89)	(-1.99)	(-2.16)	(2.61)	(2.49)
indep	0.217	0.214	0.674**	0.725**	0.345	0.515
	(1.28)	(1.27)	(2.11)	(2.16)	(0.84)	(0.99)
top5	-0.034	-0.021	-0.004	-0.023	0.212	0.228
	(-0.37)	(-0.19)	(-0.07)	(-0.39)	(1.58)	(1.56)
_cons	-0.369	-0.368	-0.092	0.024	-1.316**	-1.417**
	(-1.27)	(-1.25)	(-0.69)	(0.19)	(-2.28)	(-2.17)
N	456	456	981	981	363	363
R2	0.205	0.204	0.082	0.076	0.347	0.340
adj. R2	0.145	0.144	0.064	0.059	0.236	0.228
F	1.570	1.661	3.004	3.150	0.860	0.841

#### 5.2.5. Impact on innovation performance before and after the withdrawal of government subsidies

Policy subsidies are a common tool used to promote the healthy and orderly development of the photovoltaic industry. To examine whether such subsidies can enhance innovation performance and drive high-quality industrial development, this study assesses the effectiveness of the cohort effect in the context of subsidy policies in the photovoltaic industry. Focusing on the "531 new policy," the study compares the overall development level of the photovoltaic industry before and after the reduction in policy subsidies. As shown in Table 12, prior to the withdrawal of subsidies, the peer effect of digital transformation within the same region and industry had significant positive and negative impacts on innovation performance at the 5% and 10% levels, respectively. However, the magnitude of these effects increased after the subsidies were withdrawn, indicating that policy adjustments have contributed significantly to the improvement of innovation performance.

**Table 12:** Test results before and after policy subsidy regression.

	pre-subsidy regression		after the subsidy rollback	
	ip	ip	ip	ip
peer_a	0.029**		0.154***	
	(2.13)		(3.52)	
peer_i		-0.007*		-0.040***
		(-1.68)		(-3.65)
lev	0.063***	0.045***	0.311***	0.300***
	(3.54)	(3.99)	(4.24)	(4.17)
cashflow	0.029***	0.028***	0.395**	0.373**
	(3.87)	(3.42)	(2.44)	(2.28)
growth	-0.006***	-0.004***	0.022	0.018
	(-3.53)	(-2.65)	(0.84)	(0.69)
board	-0.027	-0.017	0.061	0.051
	(-1.52)	(-1.10)	(0.67)	(0.54)
indep	0.080	0.055	0.647**	0.755**
	(1.24)	(0.95)	(2.14)	(2.31)
top5	-0.064***	-0.055***	0.147*	0.084
	(-3.34)	(-2.62)	(1.75)	(1.06)
_cons	0.063**	0.075***	-0.744***	-0.430*
	(2.34)	(2.75)	(-2.98)	(-1.85)
N	900	900	900	900
R2	0.042	0.037	0.075	0.061
adj. R2	0.029	0.024	0.062	0.049
F	5.746	5.930	3.499	3.895

## 6. Heterogeneity analysis

### 6.1. Heterogeneity analysis based on the nature of firms’ property rights

Enterprises can be classified as state-owned or non-state-owned based on the nature of their property rights. The digital transformation peer effect varies between these two types of enterprises. A regression analysis was conducted on photovoltaic firms grouped by ownership type, with the results presented in Table 13. Columns (1) and (2) show that the peer effect of digital transformation within the same region has a significantly positive impact on innovation performance for both state-owned and non-state-owned enterprises at the 1% level. However, columns (3) and (4) reveal that the peer effect of digital transformation within the same industry has a significantly negative impact on innovation performance for non-state-owned enterprises at the 1% level, while this effect is not significant for state-owned enterprises. This may be due to non-state-owned firms, especially private enterprises, being influenced by digital transformation efforts in other firms within the same industry. However, this influence has not resulted in the expected positive effects, likely due to disadvantages in terms of government support and access to resources.

**Table 13:** Heterogeneity test results based on the nature of property rights in the photovoltaic industry.

	state-owned business		non-state enterprise	
	ip	ip	ip	ip
peer_a	0.106***		0.079***	
	(3.12)		(2.62)	
peer_i		-0.008		-0.017***
		(-0.48)		(-3.07)
lev	0.042	0.027	0.152***	0.114***
	(0.68)	(0.44)	(4.21)	(4.60)
cashflow	-0.403***	-0.357**	0.090***	0.087***
	(-2.59)	(-2.30)	(3.73)	(3.44)
growth	0.035	0.044**	-0.012***	-0.009***
	(1.56)	(2.07)	(-4.18)	(-3.18)
board	0.014	0.024	-0.044*	-0.029
	(0.21)	(0.36)	(-1.67)	(-1.09)
indep	-0.007	0.004	0.257**	0.206*
	(-0.03)	(0.01)	(2.22)	(1.96)
top5	0.044	0.087	-0.006	-0.007
	(0.52)	(0.97)	(-0.15)	(-0.18)
_cons	-0.091	-0.079	-0.047	0.022
	(-0.49)	(-0.43)	(-0.67)	(0.34)
N	477	477	1322	1322
R2	0.148	0.129	0.055	0.046
adj. R2	0.115	0.095	0.042	0.033
F	4.037	3.933	3.903	4.229

### 6.2. Heterogeneity analysis based on firm size

According to existing literature, a close correlation exists between firm size and innovation performance. On one hand, some scholars support Schumpeter’s view that the advantages of large-scale firms, such as greater capital and ease of financing, provide sustained funding for innovation. On the other hand, some scholars argue that small firms have greater advantages in technological innovation, being more flexible and quicker to respond to market changes, which may lead them to invest more in innovation to achieve progress [53]. Additionally, it has been suggested that an inverted U-shaped nonlinear relationship exists between firm size and innovation performance, where innovation performance increases with firm size up to a certain threshold, after which further growth leads to a decline in performance.

Thus, heterogeneity in firm size should be analyzed, as shown in Table 14. The results indicate that the peer effect of digital transformation within the same industry shifts from negative to positive. This suggests that smaller firms may experience negative impacts on innovation performance due to resource constraints and market competition, but as firm size increases, access to more resources and market information allows firms to better leverage the digital transformation experiences of peers, positively affecting innovation performance. Conversely, the peer effect of digital transformation within the same region shifts from positive to negative. This indicates that, for smaller firms, regional digital transformation behaviors provide valuable information and experience that promote innovation. However, as firms grow larger, increased competition and resource fragmentation within the region may negatively impact innovation performance.

**Table 14:** Heterogeneity test results based on photovoltaic firm size.

	large-scale enterprises		small-scale enterprises	
	ip	ip	ip	ip
peer_a	-0.005		0.178***	
	(-1.25)		(4.28)	
peer_i		0.004		-0.044***
		(1.20)		(-3.42)
lev	0.018***	0.021***	0.199***	0.185***
	(3.51)	(4.43)	(2.78)	(2.67)
cashflow	0.031***	0.031***	0.570*	0.560*
	(3.87)	(3.92)	(1.78)	(1.71)
growth	-0.004***	-0.005***	0.008	0.005
	(-2.65)	(-2.88)	(0.22)	(0.13)
board	0.018**	0.015**	-0.213**	-0.194*
	(2.34)	(2.09)	(-2.05)	(-1.87)
indep	-0.112***	-0.108***	1.443***	1.513***
	(-3.67)	(-3.53)	(3.63)	(3.60)
top5	-0.083***	-0.084***	0.088	0.064
	(-4.03)	(-4.06)	(1.43)	(1.00)
_cons	0.076***	0.074***	-0.248	-0.179
	(3.36)	(3.29)	(-1.32)	(-0.95)
N	925	925	875	875
R2	0.044	0.044	0.128	0.106
adj. R2	0.025	0.025	0.109	0.087
F	2.353	2.289	2.237	2.196

Managers in emerging industries should recognize these dynamic changes, carefully evaluate their firm’s size and stage of development, and consider the digital environment in their sector and region when formulating digital transformation strategies.

## 7. Conclusions and implication

### 7.1. Conclusions of the study

Based on panel data from 150 photovoltaic (PV) companies listed between 2011 and 2022, this paper empirically examines the impact of digital transformation peer effects on the innovation performance of the PV industry. The study finds the following: (1) There are significant regional and industry-specific peer effects in the digital transformation of the PV industry. Regional digital transformation peer effects positively impact the innovation performance of the PV industry, while industry peer effects have a negative impact, both of which exhibit dynamic persistence. (2) Further analysis of the transmission mechanism reveals that digital transformation peer effects primarily promote innovation performance by enhancing absorptive capacity. Moreover, the positive impact of peer effects on innovation performance becomes more pronounced with higher levels of marketization and longer executive tenure. (3) The study also incorporates an analysis of subsidy policy implementation, firm intensity types, and strategic pacing within the PV industry. The results show that after the reduction of PV policy subsidies, the peer effect of digital transformation on innovation performance is most significant for technology-intensive firms adopting analytical strategies. Before the reduction of subsidies, the peer effect was more significant for labor-intensive firms with defensive strategies. In contrast, firms with offensive strategies experience a more pronounced negative peer effect of digital transformation within the same industry. (4) Lastly, the heterogeneity analysis shows that the peer effect of digital transformation within the same industry is more pronounced for state-owned enterprises and small-scale firms in terms of its impact on innovation performance, while the regional peer effect has the opposite effect for large-scale enterprises.

### 7.2. Research implications

The findings of this paper have important implications for governments and emerging industries in leveraging digital transformation peer effects to promote the high-quality development of these industries.

1. The digital transformation peer effect should be fully leveraged, capitalizing on peer synergies within emerging industries. The study reveals significant industry and regional peer effects, with regional peer effects enhancing firms’ innovation capabilities, while industry peer effects tend to weaken them. Therefore, in the digital transformation of emerging industries, firms should learn from leading companies in the same region and maximize the benefits of geographic proximity, such as close cooperation and information exchange, to boost innovation performance. However, firms should be cautious in following industry trends, as companies within the same sector may face similar challenges and competitive pressures during the transition, potentially hindering innovation performance. The practical implications of this conclusion are as follows: the peer effects of digital transformation within the same region positively influence the innovation performance of the photovoltaic industry. Companies are encouraged to strengthen regional cooperation and share resources to foster industrial cluster development and enhance regional innovation capabilities. In contrast, peer effects of digital transformation within the same industry exert a negative impact on innovation performance. Therefore, companies should avoid homogeneous competition, promote industry self-regulation and collaboration, and stay attuned to market dynamics. These findings provide important guidance for achieving sustainable development in the photovoltaic industry amid digital transformation.
2. Maximizing the mediating and moderating pathways of digital transformation is essential for aligning internal capabilities with the external environment. The results of the transmission mechanism analysis highlight the mediating role of absorptive capacity, with marketization level and executive tenure serving as moderating factors. First, the mediating effect of absorptive capacity indicates that firms should prioritize enhancing organizational learning and innovation capabilities to fully leverage the opportunities presented by digital transformation. Second, government policy orientation must be reinforced. The marketization of emerging industries, both domestically and internationally, should be effectively advanced through stronger enforcement of market regulations, greater openness of the capital market, and a combination of "going out" and "bringing in" strategies. Additionally, enhancing the strategic coherence and execution of executive teams throughout the digital transformation process is crucial for improving innovation performance.
3. Optimizing policy support for digital transformation peer effects and fully mobilizing interactions among peer companies are essential. The analysis of government policies, firm intensity types, and strategic rhythms offers the following insights: First, in terms of policy, support such as tax incentives and financial subsidies should be provided to encourage peer firms to enhance their absorptive capacity. Second, regarding firm intensity, digital transformation strategies should be tailored to the firm’s dependence on production factors, while being mindful of the negative impacts that may arise from the peer effect in technology-intensive firms, such as excessive competition and resource waste. Third, the strategic pacing of peer firms must be adjusted. In cases of overcapacity within the industry, a strategic slowdown should be encouraged. Conversely, when conditions are favorable for emerging industries, strategic adjustments should be made promptly to seize market and policy opportunities.
4. The influence of property rights and firm size on peer effects in emerging industries is emphasized. The heterogeneity analysis reveals the varying impacts of ownership types and firm sizes. The following insights were drawn: First, non-state-owned enterprises (NSOEs) should focus on evaluating their specific conditions, developing a digital transformation strategy that aligns with their characteristics, and seeking external support and resources. Simultaneously, government and community support can facilitate NSOEs’ digital transformation by providing necessary assistance. Second, managers in emerging industries should take into account their firm’s size, stage of development, and the digital environment of their industry and region when formulating digital transformation strategies.

While this paper provides an in-depth exploration of the mechanisms through which digital transformation peer effects influence innovation performance in emerging industries, several limitations remain. Digital transformation peer effects are inherently complex and multidimensional, shaped by a variety of factors such as strategy, organization, technology, and culture. This study focuses on the influence of factors like firm type, strategic rhythm, and government subsidies. Furthermore, as the effects of digital transformation peer dynamics often exhibit a lagging nature, the analysis in this paper is limited to the impact of first-period lags on innovation performance, whereas longer-term effects require further investigation and validation. Future research could pursue the following directions: (1) Investigating the construction of peer networks in emerging industries from the perspective of industrial and supply chains to deepen the understanding of peer mechanisms, and (2) Exploring how resource optimization at the firm level can further extend the intersection of digital peer effects and economics.
